# Prediction of adult post-hemorrhagic hydrocephalus: a risk score based on clinical data

**DOI:** 10.1038/s41598-022-16577-6

**Published:** 2022-07-16

**Authors:** Zhiwen Wang, Bin Xi, Bingxiao Yu, Junhui Zhou, Min Wang, Changfeng Wang, Ruen Liu

**Affiliations:** 1 Department of Neurosurgery, Jiangxi Provincial People’s Hospital, Fenghe North Rd No. 266, Nanchang, 330000 Jiangxi Province China; 2 Department of Intensive Care Unit, Peking University Shougang Hospital, Jinyuanzhuang Road 9#, Shijingshan District, Beijing, 100144 China; 3 Department of Neurosurgery, Yuanzhou District, Yichun Second People’s Hospital, 55# East Zhongshan Road, Yichun Citye, 336000 Jiangxi Provinc China; 4 Department of Neurosurgery, Peking University People’s Hospital, 11# Xizhimen South Street, Xicheng District, Beijing, 100044 China

**Keywords:** Neuroscience, Medical research

## Abstract

There is lacking research on risk factors and prediction models associated with Post-hemorrhagic hydrocephalus (PHH). Thus, this present study aimed to analyze the risk factors of PHH and establish a risk-scoring system through a large-scale study. A retrospective study of 382 patients with intracranial hemorrhage assessed age, history and diagnosis, Glasgow coma score (GCS), and fever time. After univariate and logistic regression analysis, a risk scoring system was established according to independent risk factors and evaluated using the area under the curve (AUC). Of the 382 patients, 133 (34.8%) had PHH, 43 (11.3%) received surgical treatment. Factor classification showed that age > 60 years old [odds ratio (OR): 0.347, II = 5 points], GCS < 5 (OR: 0.09, IV = 10 points), GCS 6‒8 (OR = 0.232, III = 6 points), fever time > 9 (OR: 0.202, III = 7 points), fever time 5–9 (OR: 0.341, II = 5 points), CSF-TP x time > 14,4000 group (OR: 0.267, IV = 6 points), and CSF-TP x time 9,601‒14,400 group (OR: 0.502, III = 3 points) were independent risk factors. The result of the receiver operating characteristic (ROC) prediction showed that AUC = 0.790 (0.744‒0.836). Low-risk (IV-VII), moderate (VIII-X), and high-risk group (XI-XIII) incidence of PHH were 11.76%, 50.55%, and 70.00% (p < 0.001), respectively. The coincidence rates in the validation cohort were 26.00%, 74.07%, and 100.0% (p < 0.001), respectively. AUC value was 0.860 (0.780‒0.941). The predictive model was conducive to determining the occurrence of PHH and facilitating early intervention.

## Introduction

Post-hemorrhagic hydrocephalus (PHH) is a common complication of adult intraventricular hemorrhage (IVH), intracerebral hemorrhage (ICH), subarachnoid hemorrhage (SAH), and traumatic brain injury (TBI). Systemic intraventricular hematocele may lead to cerebrospinal fluid circulation disorders, including obstruction and absorption dysfunction^1,2^, thereby inducing ventricular dilatation, which directly affects brain metabolism and function. Moreover, PHH is always associated with poor prognosis when patients are refractory to prompt treatment. The typical symptoms include neurological dysfunction, gait abnormalities, and urinary and fecal incontinence. A proportion of patients require surgery for the improvement of their symptoms^3^, otherwise, their conditions may worsen and eventually lead to death^2^. Currently, literature reports on hydrocephalus center on the risk factors or prediction models of a disease, whereas the pathogeneses of these diseases have common characteristics, which are gradually being recognized by the public. PHH is considered an important factor in the poor prognosis of ICH^4^, IVH^5^, SAH^6^, and TBI^7^. However, predictive models for PHH are lacking. The present, large-scale study retrospectively analyzed clinical data and risk factors and established a predictive scoring system to provide support for early clinical judgment and treatment of PHH.

## Results

### Patient characteristics in the derivation cohort

A total of 382 patients were included in the study based on the inclusion and exclusion criteria. Among them, 133 patients developed PHH (including 42 cases of ventricular-peritoneal (VP) shunt surgery, two cases of cyst drainage, and one case of death). The main reason why some patients did not receive hydrocephalus shunt is because their families did not give their consent to performing the operation after comprehensive consideration. There were no statistical differences between PHH and not post-hemorrhagic hydrocephalus (nPHH) in terms of sex and diabetes. Cerebrospinal fluid total protein (CSF-TP) changed over time, and was statistically significant in both PHH and nPHH. CSF-TP x time was not only a relevant factor selected in our study, but also a key factor in PHH; the present study therefore included it in the research sequence^8^. Univariate analysis showed that the occurrence of PHH was significantly correlated with patient’s ages, GCS on admission, Disease types, days (fever duration), hypertension, treatment methods, and CSF-TP (Table 1).

**Table 1:** Clinical characteristics of patients in the derivation cohort and univariate analysis of association between potential risk factors and PHH.

Factors	PHH (n = 133)	nPHH (n = 249)	Statistics	P
**Gender**				
Male	66	125	X^2^ = 0.012	0.914
Female	67	124		
Age (years)	60.26 ± 11.03	54.65 ± 12.78	Z = -3.914	** < 0.001**
**Diabetes**				
Yes	10	12	X^2^ = 1.164	0.281
No	123	237		
**Hypertension**				
Yes	75	98	X^2^ = 10.152	**0.001**
No	58	151		
Fever time (d)	5.20 ± 3.92	3.20 ± 2.82	Z = -4.973	** < 0.001**
GCS	8.27 ± 3.30	11.13 ± 3.81	Z = -6.532	** < 0.001**
**Types**				
ICH	55	58	X^2^ = 16.72	**0.001**^**a**^
IVH	25	43		
SAH	46	135		
TBI	7	13		
**Treatment**				
EVD	72	81	X^2^ = 26.97	** < 0.001**^**b**^
Conservative	10	28		
Clip	18	88		
Embolization	24	40		
Endoscopic	9	12		
CSF-TP (mg/L)	1410.25 ± 1034.54	1201.56 ± 914.98	Z = -2.069	**0.039**
Time (days)	14.7 7 ± 6.35	10,34 ± 5.35	Z = -6.650	** < 0.001**
CSF-TP × time	15,576.45 ± 10,788.87	12,820.62 ± 11,017.47	Z = -6.265	** < 0.001**

*PHH* post-hemorrhagic hydrocephalus, *nPHH* not post-hemorrhagic hydrocephalus, *GCS* Glasgow coma score, *CSF-TP* cerebrospinal fluid total protein, *IVH* intraventricular hemorrhage, *ICH* intracerebral hemorrhage, *SAH* subarachnoid hemorrhage, *TBI* traumatic brain injury. *CSF-TP* × *time* cerebrospinal fluid total protein × time.*p < 0.05 means statistical significance.

^a^There was statistical significance between ICH and SAH.

^b^There was statistical significance between EVD and clipping, and between clipping and intervention.

After multivariate logistic regression analysis, factors with poor predictive abilities were excluded. Ultimately, age, GCS, fever time, and CSF-TP x time were determined as important predictive factors (Table 2).

**Table 2:** Multivariate analysis of that p < 0.05.

Characteristics	Category	Total patients n (%)	PHH n (%)	nPHH n (%)	OR(95%CI)	P
GCS	13–15 (I)	148 (38.7)	22 (16.5)	126 (50.6)	2.19 (1.68–2.84)	** < 0.001**
	9–12 (II)	62 (16.2)	21 (15.8)	41 (16.5)		
	6–8 (III)	142 (37.2)	70 (52.6)	72 (28.9)		
	< 6 (IV)	30 (7.9)	20 (15.0)	10 (4.0)		
Fever time	< 5 (I)	244 (63.9)	63 (47.4)	181 (72.7)	1.97 (1.31–2.94)	**0.001**
	5–9 (II)	112 (29.3)	51 (38.3)	61 (24.5)		
	> 9 (III)	26 (6.8)	19 (14.3)	7 (2.8)		
CSF-TP × time	< 4800 (I)	71 (18.6)	10 (7.5)	61 (24.5)	1.49 (1.19–1.87)	**0.001**
	4801–9600 (II)	103 (27.0)	28 (21.1)	75 (30.1)		
	9601–14,400 (III)	74 (19.4)	27 (20.3)	47 (18.9)		
	> 14,400 (IV)	134 (35.1)	68 (51.1)	66 (26.5)		
Age (years)	≤ 60 (I)	224 (58.6)	68 (51.2)	156 (62.6)	2.86 (1.70–4.82)	** < 0.001**
	> 60 (II)	158 (41.4)	65 (48.9)	93 (37.3)		

*PHH* post-hemorrhagic hydrocephalus, *nPHH* not post-hemorrhagic hydrocephalus, *GCS* Glasgow coma score, *CSF-TP* × *time* Cerebrospinal fluid total protein × time, *OR* odds ratio, *CI* confidence interval. *p < 0.05 means statistical significance.

**Figure 1:**
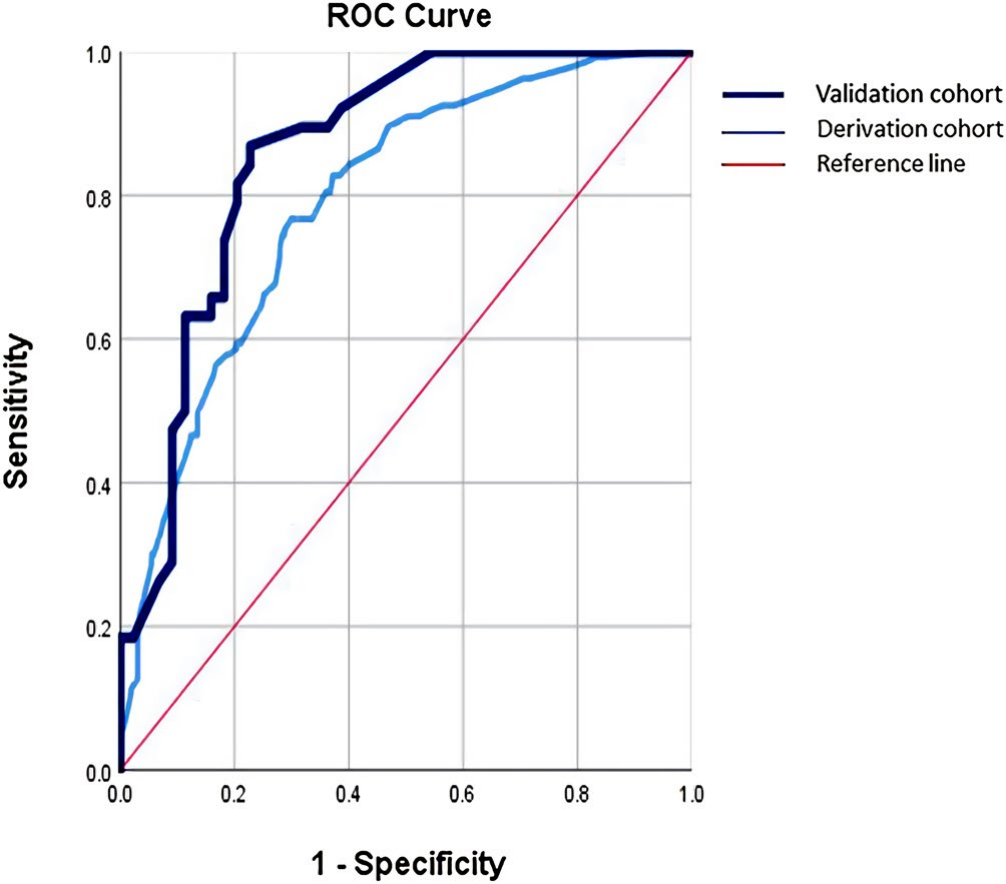
ROC curves for the scoring system in validation cohort .AUC = 0.790 (0.744–0.836), sensitivity = 0.767, specificity = 0.699. p < 0.001. Hosmer Lemeshow Test p = 0.964. ROC curves for the scoring system validation cohort. AUC = 0.860 (0.780–0.941), sensitivity = 0.868, specificity = 0.773, p < 0.001. Hosmer Lemeshow Test p = 0.244. *ROC* receiver operating characteristic, *AUC* area under the curve.

The prediction ability of derivation cohort was evaluated using ROC, and the results showed that AUC = 0.790, p < 0.001 (Fig. 1).

### Construction of the predictive scoring system

These factors were further classified according to Roman numerals. Age > 60 years, GCS < 5, GCS 6‒8, fever time > 9, fever time 5‒9, CSF-TP x time > 14,400 and 9601‒14,400 were identified as independent risk factors (Table 3).

**Table 3:** Multivariate logistic analysis of the derivation cohort and the scoring system for the prediction of PHH.

Predictors	Category	OR	95%CI	P	β-regression coefficient	Scores
GCS	< 6 (IV)	0.09	0.034–0.243	< 0.001	− 2.403	10
	6–8 (III)	0.232	0.085–0.633	0.004	− 1.463	6
Fever time	> 9 (III)	0.202	0.073–0.561	0.002	− 1.599	7
	5–9 (II)	0.341	0.121–0.956	0.041	− 1.077	5
CSF-TP × time	> 14,400 (IV)	0.267	0.119–0.602	0.001	− 1.319	6
	9601–14,400 (III)	0.502	0.271–0.93	0.028	− 0.689	3
Age (years)	> 60 (II)	0.347	0.206–0.586	< 0.001	− 1.057	5

Assignment of points to risk factors was based on a linear transformation of the corresponding β-regression coefficient: the coefficient of each variable was divided by 0.689 (the lowest β-value, corresponding to CSF-TP x time (9601–14,400), multiplied by a constant (3), and rounded to the nearest integer.

*PHH* post-hemorrhagic hydrocephalus, *GCS* Glasgow coma score, *CSF-TP* × *time* Cerebrospinal fluid total protein × time, *OR* odds ratio, *CI* confidence interval.

After the logistic regression analysis, the distribution of regression coefficients was used to assign scores to each factor. A scoring system and the corresponding PHH probability were established according to the factor grade (Table 4).

**Table 4:** A scoring system and incidence were established according to the risk levels of factors.

Grade	Score	Probability	95% CI
IV	9	0.042	0.042
V	9–11	0.074	0.042–0.112
VI	10–14	0.108	0.061–0.216
VII	12–16	0.200	0.089–0.376
VIII	13–18	0.281	0.127–0.543
IX	16–20	0.417	0.223–0.662
X	18–22	0.542	0.360–0.724
XI	21–24	0.710	0.578–0.838
XII	23–25	0.771	0.729–0.885
XIII	25–28	0.889	0.855–0.944

IV–VII: low risk; VIII–X: intermediate risk, XI–XIII: high risk, *CI *Confidence interval.

### Validation of the risk scoring system

In the following year, we validated the above scoring system. The validation cohort data included 82 patients with TBI, 38 of whom (46.34%) developed PHH. Using our risk stratification system, the PHH rates from the low-, medium-, and high-risk groups were 26.00%, 74.07%, and 100.00%, respectively. The observed incidence of PHH significantly increased as the risk score in the derivation and validation cohorts increased (p < 0.001, Supplementary material). To further evaluate the accuracy of PHH prediction, we plotted the ROC curve and calculated the AUC (Fig. 1). In the validation cohort, the AUC value of the model was 0.860 [95% confidence interval (CI), 0.780‒0.941], the sensitivity was 0.868, and the specificity was 0.773 (p < 0.001). The HL Test (p = 0.244) indicated that the risk scoring system was well differentiated and calibrated from the validation data.

## Discussion

PHH was first introduced in 1967 by Murtagh and Lehman, originally referring to the progressive expansion of neonatal IVH complicated by the ventricular system. Later, the term was used to describe hydrocephalus after SAH, TBI, and IVH^2,9^. Reportedly, the mortality rate and poor prognosis are higher in patients with PHH than in those without PHH^10,11^. Based on the time of onset, hydrocephalus is categorized as acute (< 3 days), subacute (3‒14 days), and chronic (> 14 days). PHH mainly refers to chronic hydrocephalus, and the time of onset varies between two weeks to one month^12^. Studies have pointed out that cerebral hemorrhage is closely related to hydrocephalus^13^ and hydrocephalus may occur in about 9% of patients with cerebral hemorrhage. The results of long-term follow-up studies on cerebral hemorrhage have indicated that the mortality rate of chronic hydrocephalus is similar to the overall mortality rate of cerebral hemorrhage^14^, in which 51‒89% of IVH may develop into hydrocephalus^1,2^ and 50% of IVH secondary to cerebral hemorrhage could develop into hydrocephalus^15^. The incidence of hydrocephalus occurring after subarachnoid hemorrhage varies between 15 and 37%, of which approximately 17‒21% require permanent cerebrospinal fluid (CSF) shunt operations^16^. The incidence of hydrocephalus after TBI ranges from 10.84% to 29%^17,18^. In the clinic, we often encounter such phenomena: ICH accompanied by hematoma breaking into the ventricle; IVH extending into subarachnoid space and; SAH may accumulate in the ventricle or be accompanied by a hematoma. Based on the above reasons, the analysis of PHH including the above diseases is more meaningful than the analysis of single disease complicated with hydrocephalus from the macro perspective. CSF shunt devices are permanent and therefore prone to complications, including blockage of drainage tubes and intracranial infection. Despite the commonalities between PHH and the above-mentioned diseases, there is still a lack of related literature on risk factors and prediction models of PHH, although, few studies have focused on hydrocephalus in patients under coma. To reflect the clinical characteristics of PHH more accurately, data from coma patients were collected in our study.

### Pathogenesis of PHH

The mass effect of blood clots and adhesion and obstruction mediated by intraventricular inflammation are the leading causes of hydrocephalus^18–20^. The animal model of PHH indicates that the main inducing factors of hydrocephalus are erythrocyte, hemoglobin, serum iron, and thrombin^2^, similar to the prior clinical observation results^21^. Complement 3 promotes microglia and phagocytes after red blood cell lysis, which is also a pathogenesis of PHH^22^. Numerous studies have revealed that the injury of ependymal cilia may lead to CSF disorder and hydrocephalus^23,24^ through damage models; intracerebral hemoglobin can induce the upregulation of the expression of lipocalin 2 (Lcn2), a protein related to iron treatment. Animal experiments have illustrated that the inhibition of Lcn2 is associated with a reduction in ventricular dilatation^25^. Transforming factor β1 (TGF-β1) is mainly released by platelets after hemorrhage, which can promote the synthesis of extracellular matrix proteins, further leading to subarachnoid fibrosis and eventually resulting in hydrocephalus by disrupting the flow of CSF^6,26,27^. Proteomic analysis of CSF showed that the expression levels of fibrinogen, carbonic anhydrase –I (CA-I), peroxidase-2(Prx-2), hemoglobin α and β chains, transferrin (TF), and N-terminal haptoglobin (HP) were significantly different from those of the control group^28^. In addition, interleukin (IL)-10, IL-6, IL-8, matrix metalloproteinase (MMP)-7, and MMP-9 levels were significantly elevated in the CSF protein analysis of PHH^29,30^. A new hypothesis on the cause of hydrocephalus suggests that part of the absorption and secretion of CSF occurs on the intraventricular membrane, and in some events, hydrocephalus is caused by changes in the osmotic pressure of the CSF^31–33^. In vivo studies in animals have shown that hydrocephalus is caused by an increase in the volume of CSF at high osmotic pressure and that this CSF is derived from interstitial fluid adjacent to blood vessels^34^. Krishnamurth et al. injected hypertonic dextran and fibroblast growth factor (FGF) into the ventricles of rats to simulate the increased protein content and osmotic pressure induced by blood–brain barrier rupture after IVH. These solutions increased the osmotic load and water inflow into the ventricle to normalize the osmotic gradient and successfully induced secondary hydrocephalus in a rat model^25^. Attention has been paid to the change in osmotic pressure caused by a large amount of protein accumulation in the CSF, which disrupts the osmotic balance of CSF circulation causing hydrocephalus^35^. Several studies have shown that early CSF drainage plus EVD can reduce the content of erythrocyte degradation products and CSF-TP, thereby reducing the incidence of CSF obstruction and hydrocephalus^36–38^. Given that the correlation between CSF-TP and hydrocephalus has been confirmed^39^, the present study included CSF-TP as the research object.

### Risk factors of PHH

Diringer et al. established a hydrocephalus score for the quantitative assessment of the hydrocephalus degree^15^. The significant mass effect caused by blood or hematoma in the ventricular system is the major cause of hydrocephalus, and the aggravation of hydrocephalus has been demonstrated to affect patient mortality directly^43^. The systolic blood pressure of patients with hydrocephalus on admission was significantly higher than that of patients without hydrocephalus, which was consistent with the results of the univariate analysis in this study. Multivariate analysis suggests that the degree of influence is limited; however, it is worth noting that controlling blood pressure reduces the increase of hematoma and the incidence of hydrocephalus^40^. The present study included cases of cerebral hemorrhage and SAH to explore the risk factors for PHH. The results revealed that hydrocephalus had no correlation with sex, age, bleeding location and type, and previous medical history. The risk factors included bleeding volume, intraventricular hemorrhage, and ventricular drainage. The incidence of hydrocephalus was approximately 50%, and the higher the IVH grade, the greater the risk^41^. A continuous study of 1,342 patients with cerebral hemorrhage observed that 26 had chronic hydrocephalus as a complication, and there was no statistical difference in general factors. The independent risk factors were ventricular dilatation, craniotomy, decompressive craniectomy (DC), and intracranial infection. In addition, the prognosis of patients with hydrocephalus is significantly worse than that of patients without hydrocephalus^10^. SAH retrospective analysis revealed that 36.3% of patients underwent shunt surgery after hydrocephalus, among which statistically significant factors included age, hypertension, Hunt-Hess grade, EVD operation, and intracranial infection^42,43^. Hunt-Hess grade and leukocytosis were independent risk factors for hydrocephalus. A retrospective analysis of 125 TBI cases revealed hydrocephalus in 116 patients. Statistically significant factors were related to poor prognoses, such as GCS < 8, SAH, subdural effusion, intracranial infection, annular cistern hour, coma time (> 2 months), and high fibrinogen level. Independent high-risk factors included the disappearance of the annular cistern, prolonged coma duration (> 2 months), increased plasma fibrinogen levels, and ventriculoperitoneal shunt implantation^44^. Alternatively, it was concluded that age, severe disability and low level of consciousness were independent risk factors for post-traumatic hydrocephalus (PTH)^7,17^. A national study observed that the risk of hydrocephalus in patients with TBI combined with SAH was significantly higher than that in the group without SAH, and the peak period was within 3 months after the onset of disease^45^. Due to the destruction of the anatomical and physiological integrity of the cranial cavity, the change in CSF dynamics after DC is usually considered the main cause of PTH in DC patients, and the size of craniotomy is related to hydrocephalus^18,46,47^. Continuous lumbar CSF drainage can greatly reduce the occurrence of PTH parathyroid hormone^45^.

Studies on risk factors have pointed out that craniotomy decompression and intracranial infection are independent predictors of chronic hydrocephalus^7,48^, among which larger craniotomy and decompressive craniectomy can reduce intracranial pressure but may concurrently reduce the absorption of CSF, thereby causing hydrocephalus^49,50^. Therefore, the present study excluded these two factors in the collection process when establishing the scoring system prediction model. Early prediction of PHH occurrence is conducive to assessing the patient's condition and improving prognosis.

PHH is a common complication of hemorrhagic brain disease during neurosurgery. Because of the shared characteristics of these diseases, there are many studies on single disease factors, and the influencing factors are concentrated in hematoma thickness, bleeding distribution, and bleeding volume^41,51^. This study excluded the differences in disease types and established a prediction model of PHH based on hypertension, CSF-TP over time, GCS, and fever time. Moreover, our study developed a risk scoring system to predict the occurrence of PHH. A scoring system was established according to the factor grade and statistical distribution score, which is simple and practical. In addition, the AUC value was used to evaluate the predictive ability of the model. The predictive factor was observed to be an independent risk factor for PHH. The results show the following independent risk factors: age, GCS, fever time, and CSF-TP x time. Age > 60 years, GCS < 5, GCS 6‒8, fever time > 9, fever time 5‒9 and CSF-TP x time > 14,400 and 9601‒14,400 were independent risk factors (Table 3), which are consistent with the conclusions of related reports.

This study had certain limitations. First, the sample size obtained from a single center was limited, and patients who did not undergo craniotomy within 30 days, diagnosed with intracranial infection during the course of admission, and lacking lumbar puncture results were excluded. Second, the factors included in the scoring system were limited and may affect the accuracy of the scoring system. Third, there may be recall and selection biases, and it is not excluded that some factors are influencing factors of PHH. Fourth, although CSF-TP as clinical evidence can indirectly suggest changes in colloid osmotic pressure in cerebrospinal fluid. However, direct colloid osmotic pressure test results of cerebrospinal fluid are lacking. Fifth, information errors in evaluating patients may lead to the loss of some patients with hydrocephalus and a lack of long-term prognosis evaluation. However, early judgment and intervention are important in reducing the occurrence of PHH. Therefore, a scoring system was developed to increase the general applicability of the model for popularization and practicality in the clinic.

In conclusion, the pathogenesis of PHH is a complex clinical-pathological process. Numerous studies have been conducted regarding the related mechanisms, indicating that many factors are involved in its pathogenesis. Moreover, early prediction of PHH occurrence is conducive to assessing the patients’ condition and prognosis improvement. Furthermore, to the best of our knowledge, our study is the first to propose a risk scoring system for PHH. We observed that age, GCS, fever time, and CSF-TP x time were important risk factors for predicting PHH, which can positively contribute to the early detection and treatment of PHH by clinicians.

## Methods

### Case selection

Data regarding related cases (including ICH, IVH, SAH, and TBI) admitted to the Department of Neurosurgery of Jiangxi Provincial People's Hospital from January 2013 to January 2022 were collected retrospectively. We excluded patients without cerebrospinal fluid test results, decompressive craniectomy, and intracranial infection to explore the same characteristics of PHH. The objectives of this study were to analyze the risk factors for hemorrhagic brain diseases complicated by PHH and establish a comprehensive prediction model. This study was conducted in strict accordance with the 2013 Declaration of Helsinki guidelines. The cases were selected according to the following criteria:

### Inclusion criteria

(1) A definitive diagnosis of hemorrhagic brain disease (TBI, SAH, IVH, ICH, etc.) confirmed by computed tomography (CT).

(2) Cerebrospinal fluid test results were available within 30 days of the duration of the disease.

(3) A traceable course of the disease for more than 2 weeks from admission.

### Exclusion criteria

1. A prior diagnosis of hematological diseases, brain tumors, or hydrocephalus.
2. A diagnosis of intracranial infection during the course of the disease.
3. Craniotomy and decompression of the bone flap were performed later on (not repaired within 30 days).
4. Complications of the liver, kidney, gastrointestinal, respiratory, and cardiovascular systems, and other diseases that seriously affect safety evaluation.
5. Complications with serious infectious diseases.
6. Complications with coagulation dysfunction and patients on long-term anticoagulants
7. Pregnant and lactating women.

### Treatments

All patients received standardized nursing and neurosurgical treatments. Our department has a well-trained, professional team that strictly follows the state guidelines on neurosurgical treatment. After admission, the patients underwent cranial CT, routine blood and biochemical tests, other relevant examinations, and a physical examination by the neurosurgery specialist. The onset and medical history of the patients were precisely recorded.

### Diagnosis of PHH

The diagnosis of PHH was mainly based on imaging results and clinical symptoms. Patients with hydrocephalus gradually developed characteristic symptoms of increased intracranial pressure (headache, nausea/vomiting, altered consciousness) and signs (such as low level of consciousness and papiloedema of the optic nerve).The specific criteria are as follows: (a) The Evans index was greater than 0.3; (b) An enlargement of the anterior horn, temporal horn, third ventricle of the lateral ventricle, and periventricular interstitial edema^52^; (c) Neurocognitive dysfunction in conscious patients (such as depression, difficulty in decision-making, memory or language disorder, physical activity disorders including walking instability and ataxia, and dysuria); (d) No improvement or worsening of consciousness in patients under coma (with images indicating hydrocephalus).

### Data collection

Basic information, including age, sex, diagnosis, and Glasgow Coma Score (GCS), among others, were obtained from the inpatient information management system of the hospital. Medical history (such as history of hypertension, diabetes, illicit drug use, and personal history), treatment information (conservative treatment, external ventricular drain (EVD), embolization, and endoscopic treatment), fever time (the total time when the body temperature is ≥ 38.5 °C), cerebrospinal fluid biochemical results and time (from the onset of the disease to the obtainment of CSF-TP after lumbar puncture) were collected. Relevant studies suggest that inflammation and fever are related to hydrocephalus^53,54^. A large accumulation of various proteins in the cerebrospinal fluid will change osmotic pressure and cause hydrocephalus, which has been verified in animal models^35,53^, and the CSF-TP of hydrocephalus patients is higher than that of the normal group^39^. In our previous study, we observed for the first time that fever time and CSF-TP were two important factors in the statistical results^8^. The prognostic information of the selected patients was followed up within 6 months from their discharge. PHH diagnosis was based on the diagnosis when the patient was discharged from the hospital, re-examination of symptoms and examination results, re-admission examination, and treatment information. The collection of information and statistical analyses were carried out by professional personnel.

After a univariate analysis of all factors, those with p < 0.05 were selected for multivariate logistic regression analysis, and important risk factors were selected to establish the prediction model. The scoring system for the prediction model was obtained according to the distribution of the β regression coefficient values for each factor. The factor grade or the score grade and the corresponding prediction probability were established according to the above score, which is convenient for clinical use.

### Statistical analysis

SPSS software (version 26.0, SPSS Inc., Chicago, Illinois, USA) was used for the statistical analysis. The statistical tests were bilateral. Categorical variables were expressed as count and percentage (%) and continuous variables were expressed as mean (x) ± standard deviation (SD). Univariate analysis was performed by t-test, Chi-square test, or U test. A p value < 0.05 was considered statistically significant. In the univariate analysis, variables related to PHH (p < 0.05) were included in the logistic regression model to determine the independent predictors of PHH. Factors with p < 0.05 were grouped into logistic regression analysis. The receiver operating characteristic (ROC) curve and area under the curve (AUC) were used to evaluate the discrimination ability of the scoring system, which was divided into good to excellent (AUC > 0.8), medium (AUC 0.7‒0.8), and low (AUC 0.6‒0.7). The Hosmer‒Lemeshow (HL) test was used to evaluate the calibration ability of the model.

### Ethics approval

The study was approved by the biomedical Ethics Committee of Jiangxi Provincial People's Hospital (No: 2022-018) and the informed consent was waived by the biomedical Ethics Committee of Jiangxi Provincial People's Hospital. In accordance with the requirements of national legislation and institutions, written informed consent of patients or their families is not required for this study. All methods were carried out in accordance with relevant guidelines and regulations.

## Supplementary Information

Supplementary Information 1.

Supplementary Information 2.

Supplementary Information 3.

Supplementary Information 4.

## Data Availability

The original contributions presented in the study are included in the article/Supplementary Material, further inquiries can be directed to the corresponding author/s.

## Abbreviations

PHH: Post-hemorrhagic hydrocephalus
GCS: Glasgow coma score
AUC: Area under curve
CSF-TP: Cerebrospinal fluid total protein
OR: Odds ratio
CI: Confidence interval
IVH: Intraventricular hemorrhage
ICH: Intracerebral hemorrhage
SAH: Subarachnoid hemorrhage
TBI: Traumatic brain injury
CT: Computed tomography
EVD: External ventricular drain
SD: Sstandard deviation
ROC: Receiver operating characteristic
HL: Hosmer–Lemeshow
VP: Ventricular-peritoneal
nPHH: Not post-hemorrhagic hydrocephalus
CSF: Cerebrospinal fluid
Lcn2: Lipocalin 2
TGF-β1: Transforming factor-β1
CA-I: Carbonic anhydrase-I
Prx-2: Peroxidase-2
TF: Transferrin
HP: Haptoglobin
IL: Interleukin
MMP: Matrix metallo protein
FGF: Fibroblast growth factor
DC: Decompressive craniectomy
PTH: Post-traumatic hydrocephalus
