# Supplementary material for: Prediction of adult post-hemorrhagic hydrocephalus: a risk score based on clinical data
Source: Sci Rep. 2022 Jul 16;12:12213. doi: 10.1038/s41598-022-16577-6 (PMC9288433; doi:10.1038/s41598-022-16577-6)

**Prediction of Adult Post-hemorrhagic Hydrocephalus: A Risk Score Based on Clinical Data**

**Supplementary files**

Supplementary table 1. Select the important factors after single factor analysis (p < 0.05) to carry out multiple factors. *GCS Glasgow coma score, CSF-TP x time Cerebrospinal fluid total protein x time. OR odds ratio, CI Confidence interval.*

| **Predictors** | **OR** | **95%CI** | **P** |
| --- | --- | --- | --- |
| Age (yrs) | 1.067 | 1.038-1.096 | **<0.001** |
| Treatment | 1.691 | 0.414-7.428 | 0.528 |
| GCS | 0.853 | 0.786-0.927 | **<0.001** |
| Types | 0.780 | 0.189-3.291 | 0.600 |
| Hypertension | 1.165 | 0.682-1.992 | 0.575 |
| Days | 1.175 | 1.081-1.278 | **<0.001** |
| CSF-TP x time | 1.000 | 1.000-1.000 | **0.001** |

Supplementary table 2. Observed incidence of PHH in the derivation and validation cohorts according to risk grade. *PHH Post-hemorrhagic hydrocephalus, nPHH not Post-hemorrhagic hydrocephalus.*

| **Risk caregory** | **Derivation cohort (n = 382)** | | **Validation cohort (n=82)** | |
| --- | --- | --- | --- | --- |
|  | **nPHH (%)** | **PHH (%)** | **nPHH (%)** | **PHH (%)** |
| Low (IV - VII) | 150 | 20 (11.76) | 37 | 13 (26.00) |
| Intermediate (VIII - X) | 90 | 92 (50.55) | 7 | 20 (74.07) |
| High (XI - XIII) | 9 | 21 (70.00) | 0 | 5 (100.00) |
| χ2 for trend | 76.02 | | 22.46 | |
| p value for trend | **< 0.001** | | **< 0.001** | |

Supplementary figure 1. Lnear distribution of CSF-TP in PHH group and nPHH group.


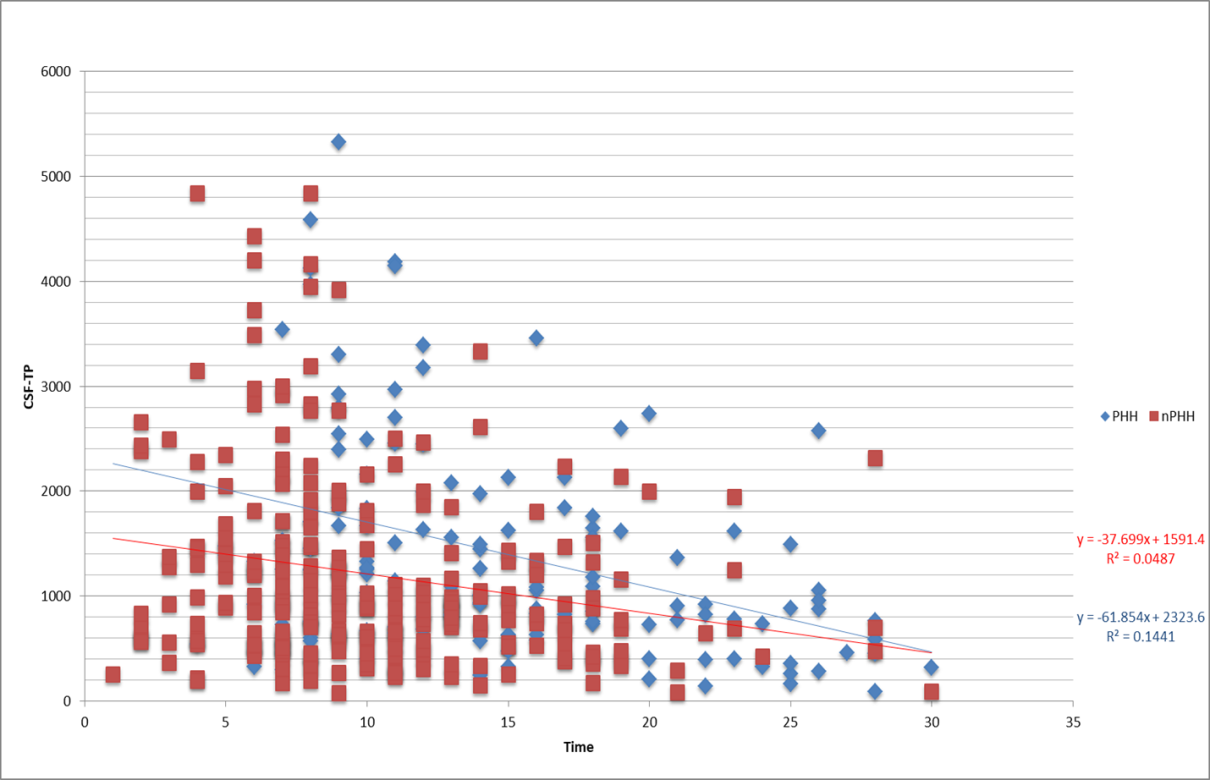


Supplementary figure 2. PHH occurred in PHH group and nPHH group according to time grouping and CSF-TP grouping.


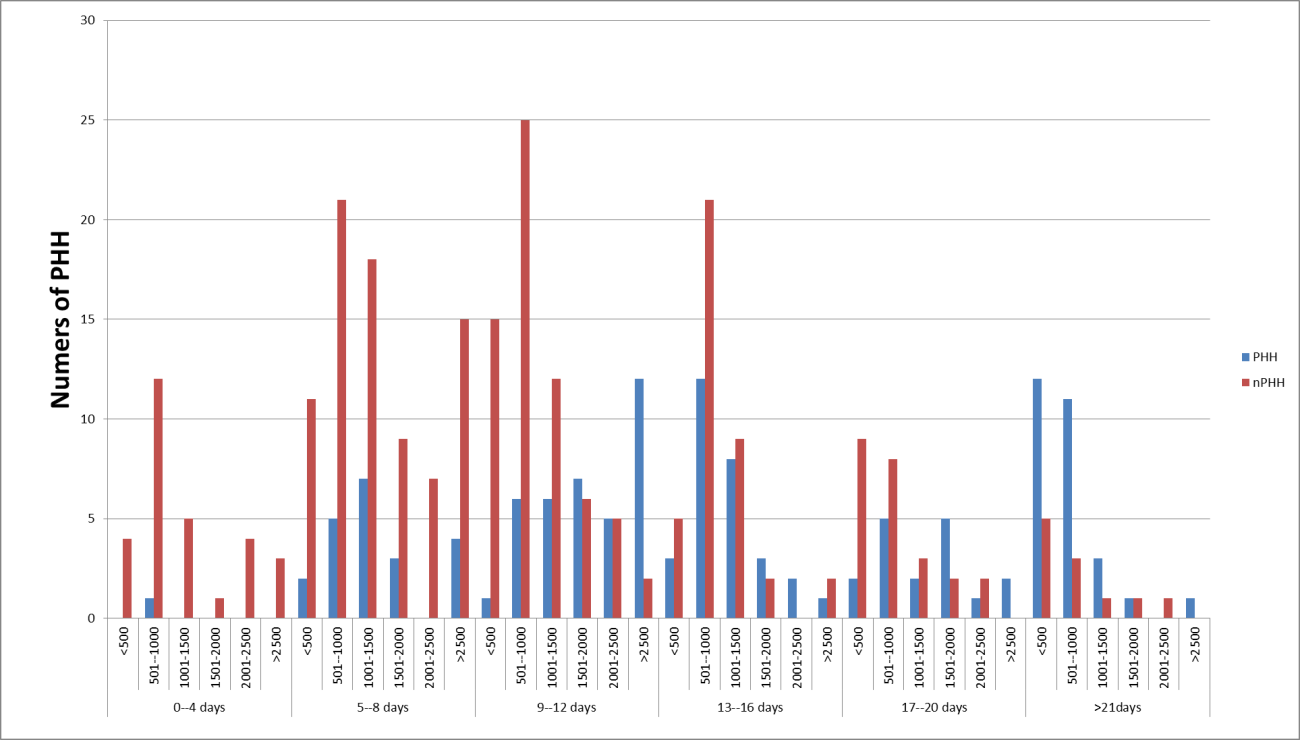

Supplement: Supplementary file 2 — Supplementary Information 2. [file 41598_2022_16577_MOESM2_ESM.docx]
