# Supplementary material for: Prediction of adult post-hemorrhagic hydrocephalus: a risk score based on clinical data
Source: Sci Rep. 2022 Jul 16;12:12213. doi: 10.1038/s41598-022-16577-6 (PMC9288433; doi:10.1038/s41598-022-16577-6)
Supplement: Supplementary file 3 — Supplementary Information 3. [file 41598_2022_16577_MOESM3_ESM.docx]

| Supplementary files | Title and legend |
| --- | --- |
| Supplementary table 1 | Select the important factors after single factor analysis (p < 0.05) to carry out multiple factors. |
| Supplementary table 2 | Observed incidence of PHH in the derivation and validation cohorts according to risk grade. |
| Supplementary figure 1 | Linear distribution of CSF-TP in PHH group and nPHH group |
| Supplementary figure 2 | PHH occurred in PHH group and nPHH group according to time grouping and CSF-TP grouping. |

**Supplementary files—table title and legend**
